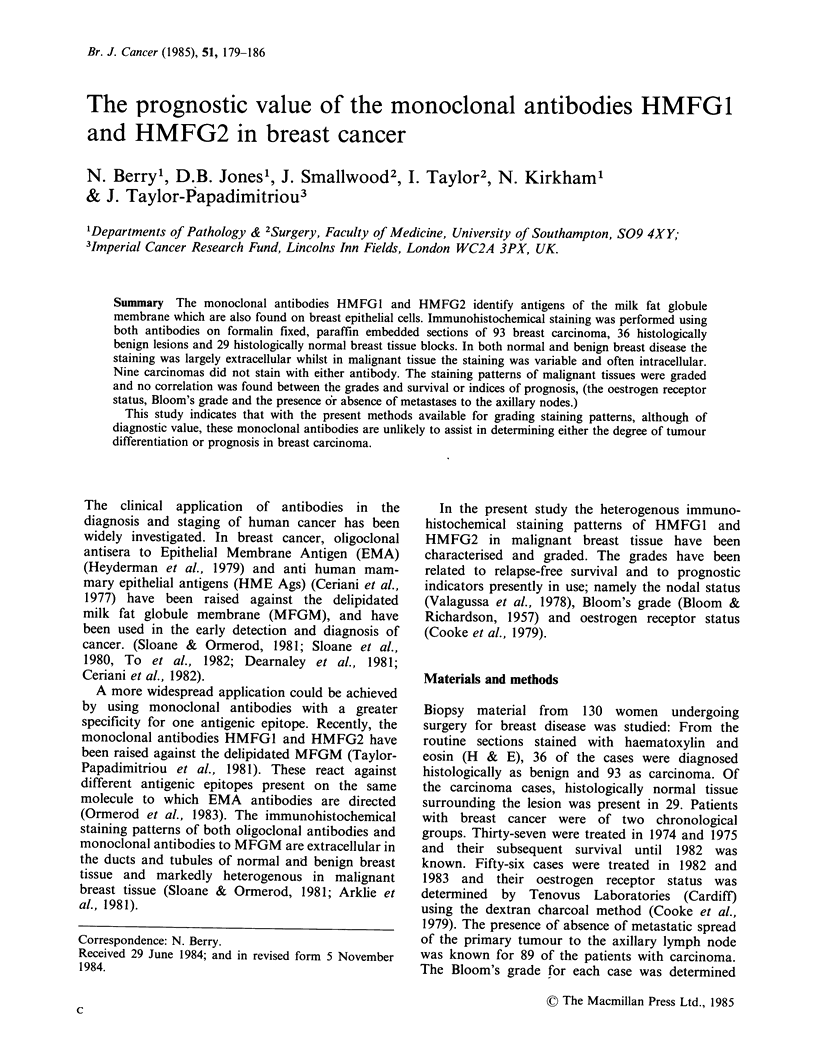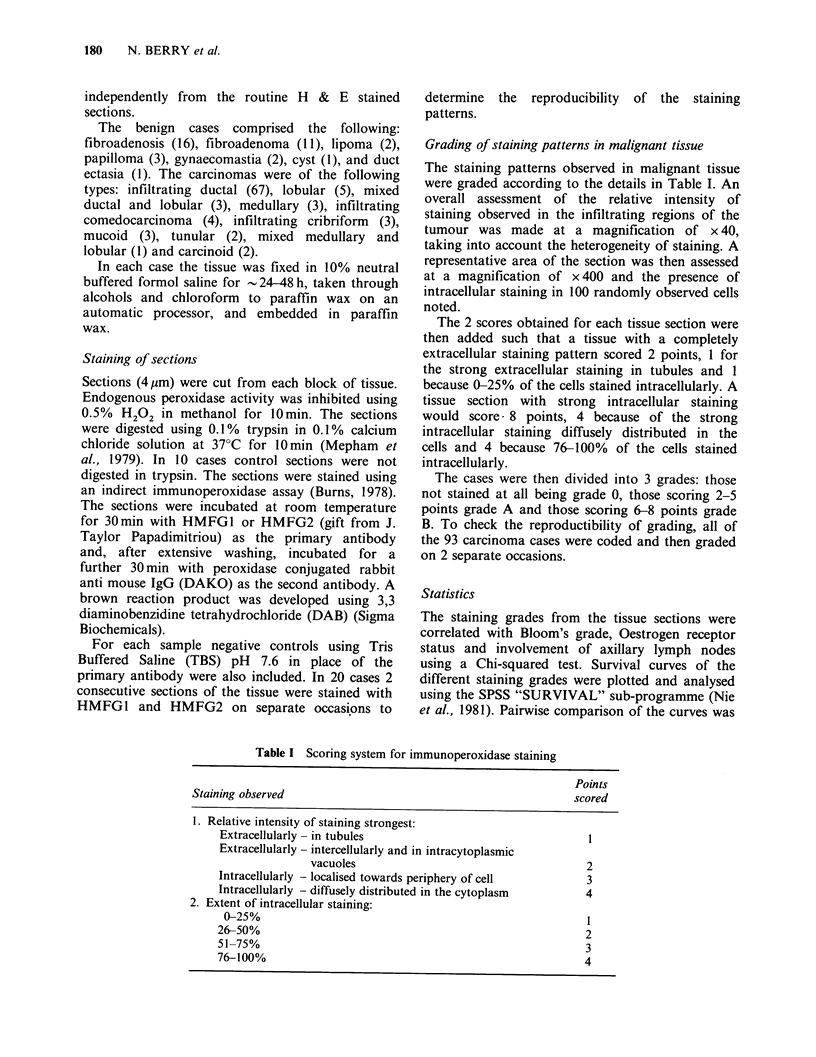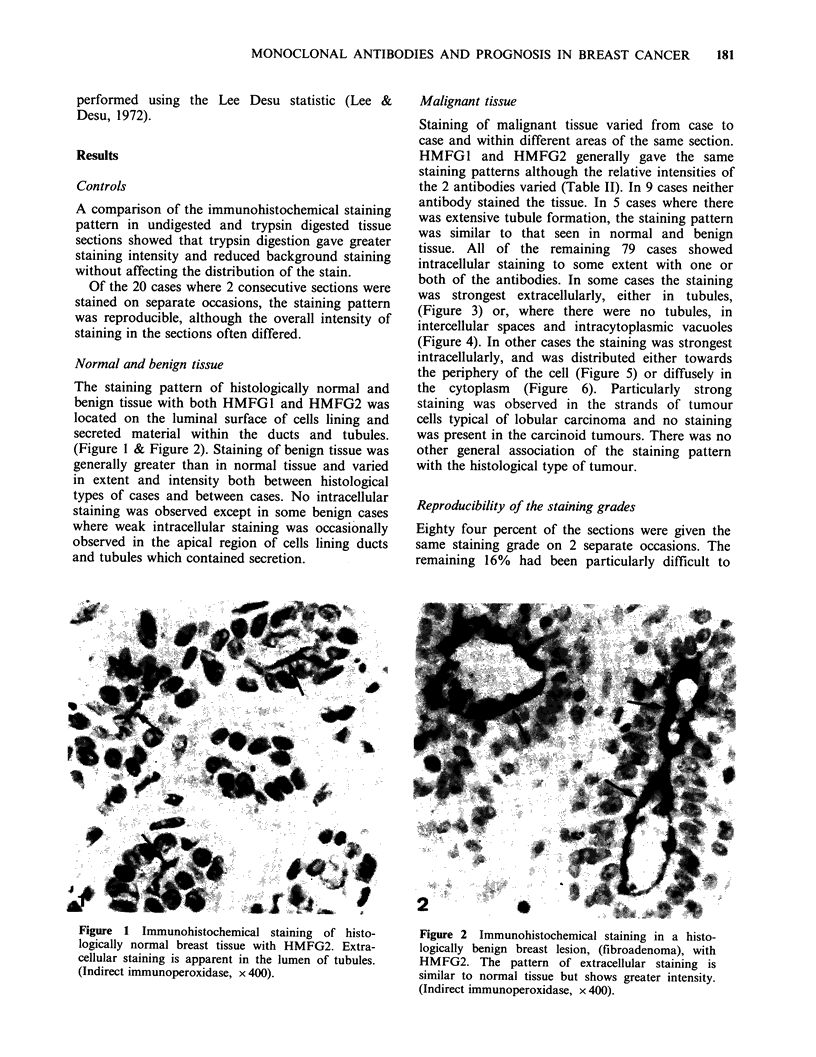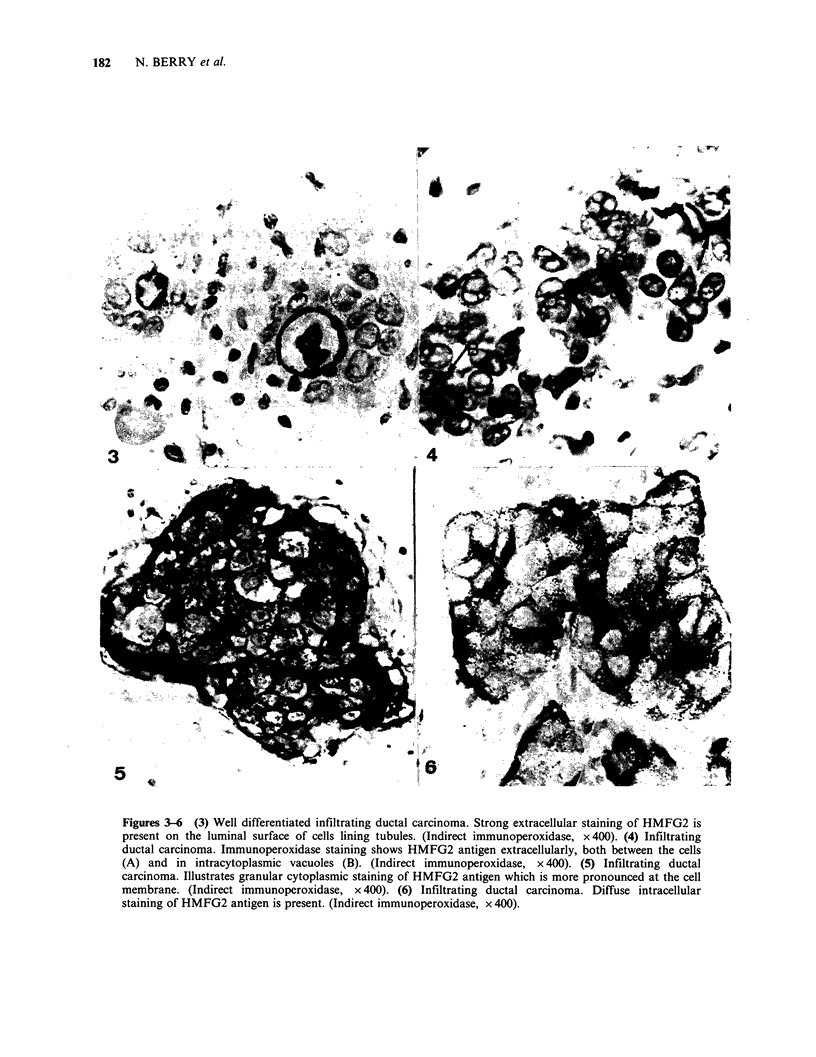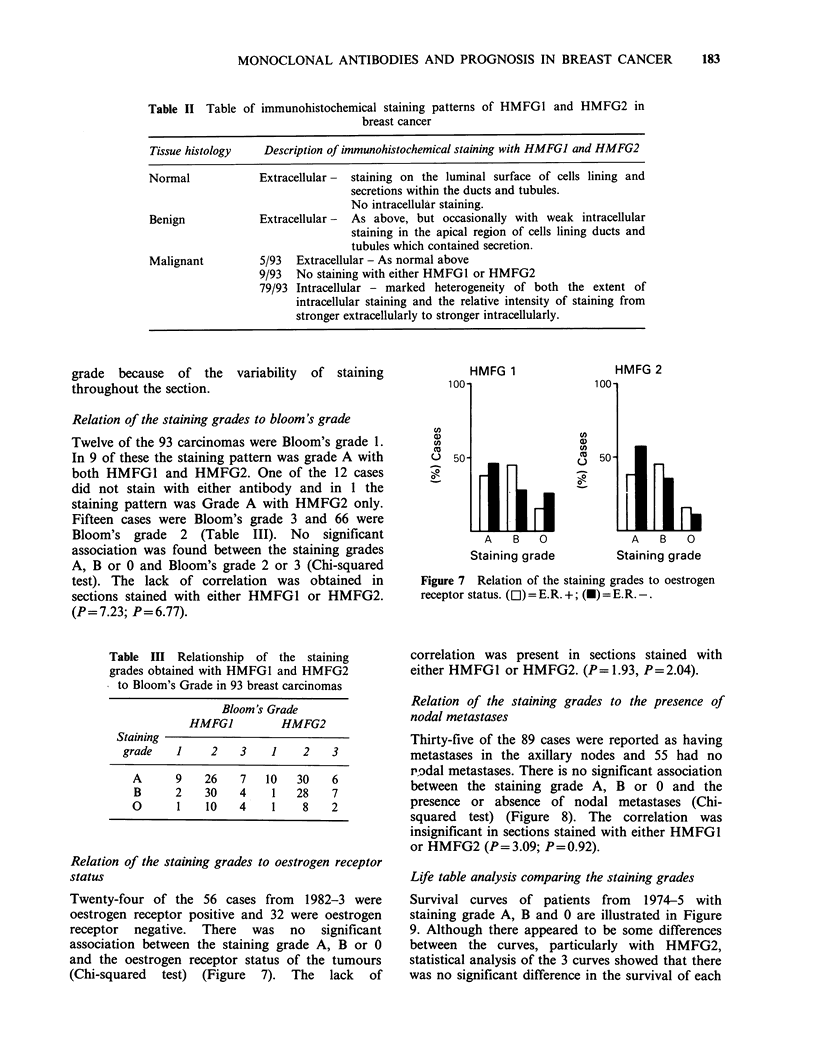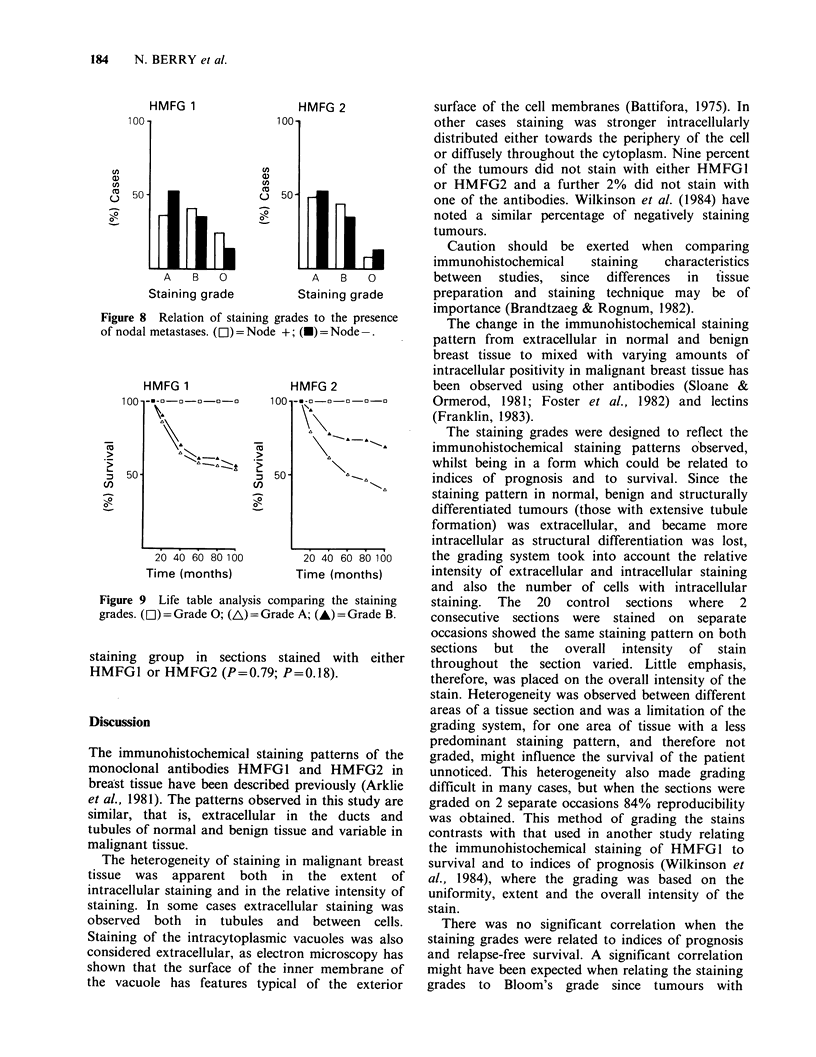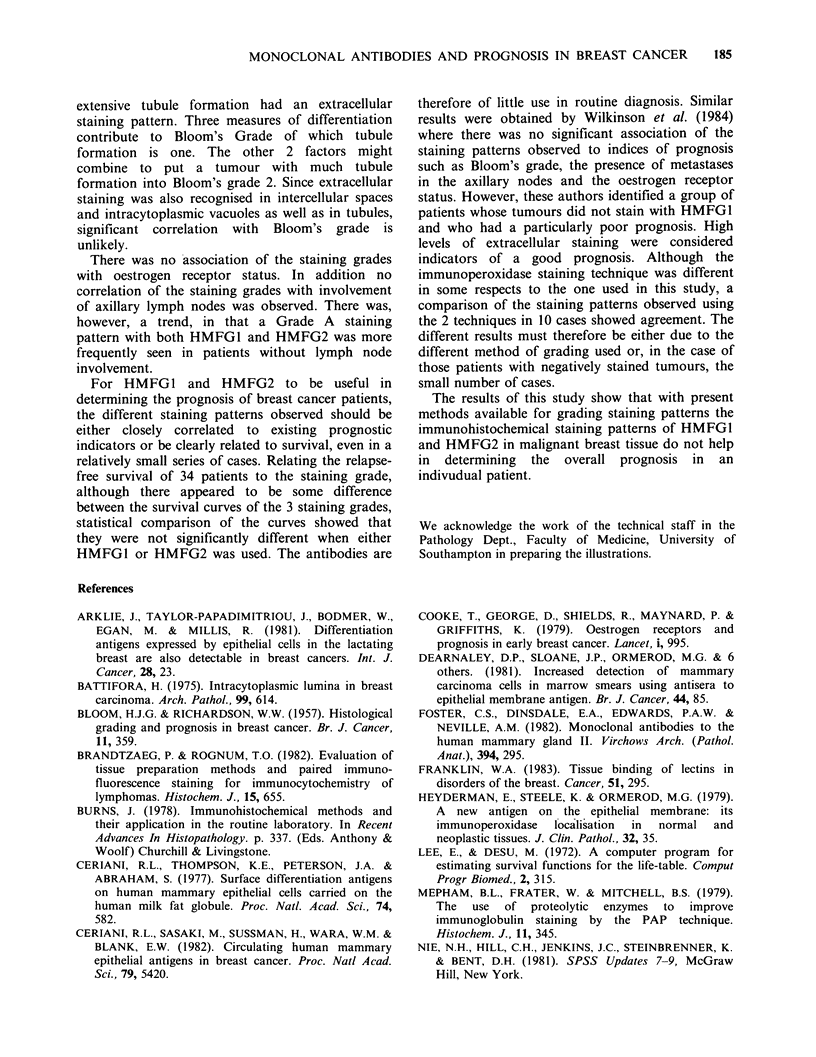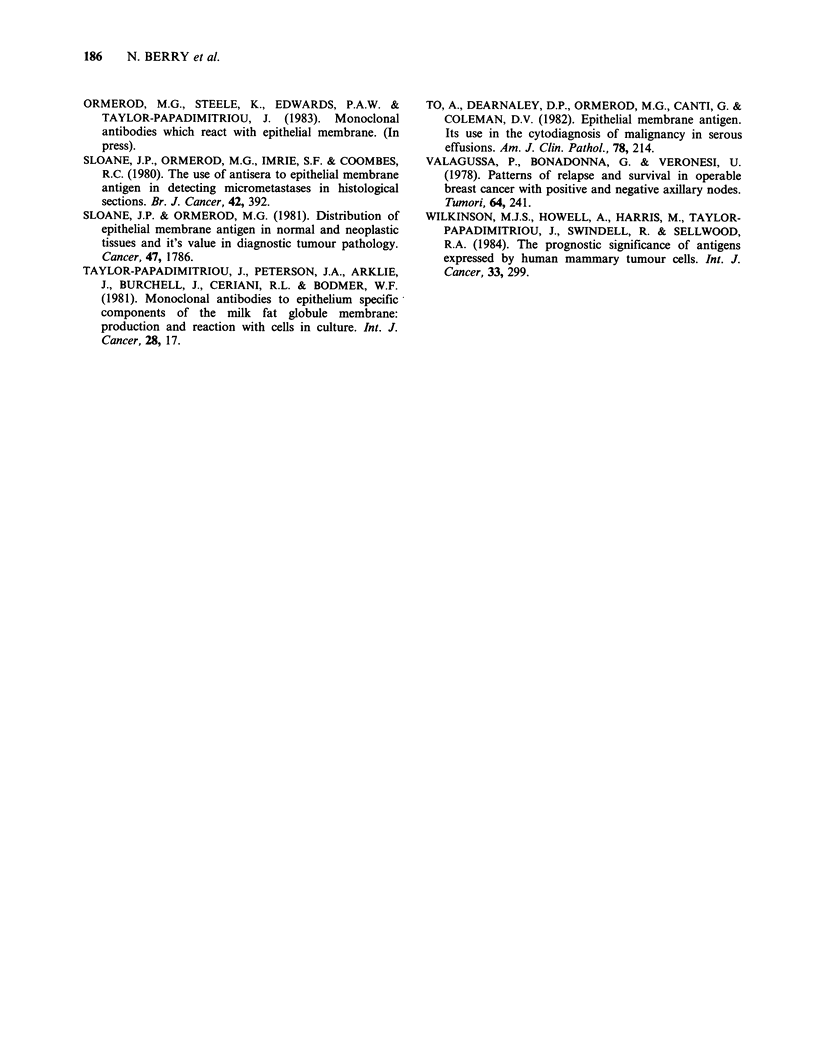# The prognostic value of the monoclonal antibodies HMFG1 and HMFG2 in breast cancer.

**DOI:** 10.1038/bjc.1985.27

**Published:** 1985-02

**Authors:** N. Berry, D. B. Jones, J. Smallwood, I. Taylor, N. Kirkham, J. Taylor-Papadimitriou

## Abstract

**Images:**


*Br. J. Cancer* (1985), **51**, 179–186

### The prognostic value of the monoclonal antibodies HMFG1 and HMFG2 in breast cancer

N. Berry[1], D.B. Jones[1], J. Smallwood[2], I. Taylor[2], N. Kirkham[1] & J. Taylor-Papadimitriou[3]

[1]*Departments of Pathology &* [2]*Surgery, Faculty of Medicine, University of Southampton, SO9 4XY;* [3]*Imperial Cancer Research Fund, Lincolns Inn Fields, London WC2A 3PX, UK.*

**Summary** The monoclonal antibodies HMFG1 and HMFG2 identify antigens of the milk fat globule membrane which are also found on breast epithelial cells. Immunohistochemical staining was performed using both antibodies on formalin fixed, paraffin embedded sections of 93 breast carcinoma, 36 histologically benign lesions and 29 histologically normal breast tissue blocks. In both normal and benign breast disease the staining was largely extracellular whilst in malignant tissue the staining was variable and often intracellular. Nine carcinomas did not stain with either antibody. The staining patterns of malignant tissues were graded and no correlation was found between the grades and survival or indices of prognosis, (the oestrogen receptor status, Bloom's grade and the presence or absence of metastases to the axillary nodes.)

This study indicates that with the present methods available for grading staining patterns, although of diagnostic value, these monoclonal antibodies are unlikely to assist in determining either the degree of tumour differentiation or prognosis in breast carcinoma.

The clinical application of antibodies in the diagnosis and staging of human cancer has been widely investigated. In breast cancer, oligoclonal antisera to Epithelial Membrane Antigen (EMA) (Heyderman *et al.*, 1979) and anti human mammary epithelial antigens (HME Ags) (Ceriani *et al.*, 1977) have been raised against the delipidated milk fat globule membrane (MFGM), and have been used in the early detection and diagnosis of cancer. (Sloane & Ormerod, 1981; Sloane *et al.*, 1980, To *et al.*, 1982; Dearnaley *et al.*, 1981; Ceriani *et al.*, 1982).

A more widespread application could be achieved by using monoclonal antibodies with a greater specificity for one antigenic epitope. Recently, the monoclonal antibodies HMFG1 and HMFG2 have been raised against the delipidated MFGM (Taylor-Papadimitriou *et al.*, 1981). These react against different antigenic epitopes present on the same molecule to which EMA antibodies are directed (Ormerod *et al.*, 1983). The immunohistochemical staining patterns of both oligoclonal antibodies and monoclonal antibodies to MFGM are extracellular in the ducts and tubules of normal and benign breast tissue and markedly heterogenous in malignant breast tissue (Sloane & Ormerod, 1981; Arklie *et al.*, 1981).

Correspondence: N. Berry.
Received 29 June 1984; and in revised form 5 November 1984.

In the present study the heterogenous immunohistochemical staining patterns of HMFG1 and HMFG2 in malignant breast tissue have been characterised and graded. The grades have been related to relapse-free survival and to prognostic indicators presently in use; namely the nodal status (Valagussa *et al.*, 1978), Bloom's grade (Bloom & Richardson, 1957) and oestrogen receptor status (Cooke *et al.*, 1979).

### Materials and methods

Biopsy material from 130 women undergoing surgery for breast disease was studied: From the routine sections stained with haematoxylin and eosin (H & E), 36 of the cases were diagnosed histologically as benign and 93 as carcinoma. Of the carcinoma cases, histologically normal tissue surrounding the lesion was present in 29. Patients with breast cancer were of two chronological groups. Thirty-seven were treated in 1974 and 1975 and their subsequent survival until 1982 was known. Fifty-six cases were treated in 1982 and 1983 and their oestrogen receptor status was determined by Tenovus Laboratories (Cardiff) using the dextran charcoal method (Cooke *et al.*, 1979). The presence of absence of metastatic spread of the primary tumour to the axillary lymph node was known for 89 of the patients with carcinoma. The Bloom's grade for each case was determined

© The Macmillan Press Ltd., 1985

independently from the routine H & E stained sections.

The benign cases comprised the following: fibroadenosis (16), fibroadenoma (11), lipoma (2), papilloma (3), gynaecomastia (2), cyst (1), and duct ectasia (1). The carcinomas were of the following types: infiltrating ductal (67), lobular (5), mixed ductal and lobular (3), medullary (3), infiltrating comedocarcinoma (4), infiltrating cribriform (3), mucoid (3), tunular (2), mixed medullary and lobular (1) and carcinoid (2).

In each case the tissue was fixed in 10% neutral buffered formol saline for ~24–48 h, taken through alcohols and chloroform to paraffin wax on an automatic processor, and embedded in paraffin wax.

*Staining of sections*

Sections (4 μm) were cut from each block of tissue. Endogenous peroxidase activity was inhibited using 0.5% $H_2O_2$ in methanol for 10 min. The sections were digested using 0.1% trypsin in 0.1% calcium chloride solution at 37°C for 10 min (Mepham *et al.*, 1979). In 10 cases control sections were not digested in trypsin. The sections were stained using an indirect immunoperoxidase assay (Burns, 1978). The sections were incubated at room temperature for 30 min with HMFG1 or HMFG2 (gift from J. Taylor Papadimitriou) as the primary antibody and, after extensive washing, incubated for a further 30 min with peroxidase conjugated rabbit anti mouse IgG (DAKO) as the second antibody. A brown reaction product was developed using 3,3 diaminobenzidine tetrahydrochloride (DAB) (Sigma Biochemicals).

For each sample negative controls using Tris Buffered Saline (TBS) pH 7.6 in place of the primary antibody were also included. In 20 cases 2 consecutive sections of the tissue were stained with HMFG1 and HMFG2 on separate occasions to determine the reproducibility of the staining patterns.

*Grading of staining patterns in malignant tissue*

The staining patterns observed in malignant tissue were graded according to the details in Table I. An overall assessment of the relative intensity of staining observed in the infiltrating regions of the tumour was made at a magnification of ×40, taking into account the heterogeneity of staining. A representative area of the section was then assessed at a magnification of ×400 and the presence of intracellular staining in 100 randomly observed cells noted.

The 2 scores obtained for each tissue section were then added such that a tissue with a completely extracellular staining pattern scored 2 points, 1 for the strong extracellular staining in tubules and 1 because 0–25% of the cells stained intracellularly. A tissue section with strong intracellular staining would score 8 points, 4 because of the strong intracellular staining diffusely distributed in the cells and 4 because 76–100% of the cells stained intracellularly.

The cases were then divided into 3 grades: those not stained at all being grade 0, those scoring 2–5 points grade A and those scoring 6–8 points grade B. To check the reproductibility of grading, all of the 93 carcinoma cases were coded and then graded on 2 separate occasions.

*Statistics*

The staining grades from the tissue sections were correlated with Bloom's grade, Oestrogen receptor status and involvement of axillary lymph nodes using a Chi-squared test. Survival curves of the different staining grades were plotted and analysed using the SPSS "SURVIVAL" sub-programme (Nie *et al.*, 1981). Pairwise comparison of the curves was

**Table I** Scoring system for immunoperoxidase staining

| *Staining observed* | *Points scored* |
|---|---|
| 1. Relative intensity of staining strongest: | |
| Extracellularly – in tubules | 1 |
| Extracellularly – intercellularly and in intracytoplasmic vacuoles | 2 |
| Intracellularly – localised towards periphery of cell | 3 |
| Intracellularly – diffusely distributed in the cytoplasm | 4 |
| 2. Extent of intracellular staining: | |
| 0–25% | 1 |
| 26–50% | 2 |
| 51–75% | 3 |
| 76–100% | 4 |

performed using the Lee Desu statistic (Lee & Desu, 1972).

### Results

### *Controls*

A comparison of the immunohistochemical staining pattern in undigested and trypsin digested tissue sections showed that trypsin digestion gave greater staining intensity and reduced background staining without affecting the distribution of the stain.

Of the 20 cases where 2 consecutive sections were stained on separate occasions, the staining pattern was reproducible, although the overall intensity of staining in the sections often differed.

### *Normal and benign tissue*

The staining pattern of histologically normal and benign tissue with both HMFG1 and HMFG2 was located on the luminal surface of cells lining and secreted material within the ducts and tubules. (Figure 1 & Figure 2). Staining of benign tissue was generally greater than in normal tissue and varied in extent and intensity both between histological types of cases and between cases. No intracellular staining was observed except in some benign cases where weak intracellular staining was occasionally observed in the apical region of cells lining ducts and tubules which contained secretion.

### *Malignant tissue*

Staining of malignant tissue varied from case to case and within different areas of the same section. HMFG1 and HMFG2 generally gave the same staining patterns although the relative intensities of the 2 antibodies varied (Table II). In 9 cases neither antibody stained the tissue. In 5 cases where there was extensive tubule formation, the staining pattern was similar to that seen in normal and benign tissue. All of the remaining 79 cases showed intracellular staining to some extent with one or both of the antibodies. In some cases the staining was strongest extracellularly, either in tubules, (Figure 3) or, where there were no tubules, in intercellular spaces and intracytoplasmic vacuoles (Figure 4). In other cases the staining was strongest intracellularly, and was distributed either towards the periphery of the cell (Figure 5) or diffusely in the cytoplasm (Figure 6). Particularly strong staining was observed in the strands of tumour cells typical of lobular carcinoma and no staining was present in the carcinoid tumours. There was no other general association of the staining pattern with the histological type of tumour.

### *Reproducibility of the staining grades*

Eighty four percent of the sections were given the same staining grade on 2 separate occasions. The remaining 16% had been particularly difficult to

**Figure 1** Immunohistochemical staining of histologically normal breast tissue with HMFG2. Extracellular staining is apparent in the lumen of tubules. (Indirect immunoperoxidase, ×400).

**Figure 2** Immunohistochemical staining in a histologically benign breast lesion, (fibroadenoma), with HMFG2. The pattern of extracellular staining is similar to normal tissue but shows greater intensity. (Indirect immunoperoxidase, ×400).

**Figures 3–6** **(3)** Well differentiated infiltrating ductal carcinoma. Strong extracellular staining of HMFG2 is present on the luminal surface of cells lining tubules. (Indirect immunoperoxidase, ×400). **(4)** Infiltrating ductal carcinoma. Immunoperoxidase staining shows HMFG2 antigen extracellularly, both between the cells (A) and in intracytoplasmic vacuoles (B). (Indirect immunoperoxidase, ×400). **(5)** Infiltrating ductal carcinoma. Illustrates granular cytoplasmic staining of HMFG2 antigen which is more pronounced at the cell membrane. (Indirect immunoperoxidase, ×400). **(6)** Infiltrating ductal carcinoma. Diffuse intracellular staining of HMFG2 antigen is present. (Indirect immunoperoxidase, ×400).

**Table II** Table of immunohistochemical staining patterns of HMFG1 and HMFG2 in breast cancer

| *Tissue histology* | *Description of immunohistochemical staining with HMFG1 and HMFG2* | |
|---|---|---|
| Normal | Extracellular – | staining on the luminal surface of cells lining and secretions within the ducts and tubules. No intracellular staining. |
| Benign | Extracellular – | As above, but occasionally with weak intracellular staining in the apical region of cells lining ducts and tubules which contained secretion. |
| Malignant | 5/93 | Extracellular – As normal above |
| | 9/93 | No staining with either HMFG1 or HMFG2 |
| | 79/93 | Intracellular – marked heterogeneity of both the extent of intracellular staining and the relative intensity of staining from stronger extracellularly to stronger intracellularly. |

grade because of the variability of staining throughout the section.

*Relation of the staining grades to bloom's grade*

Twelve of the 93 carcinomas were Bloom's grade 1. In 9 of these the staining pattern was grade A with both HMFG1 and HMFG2. One of the 12 cases did not stain with either antibody and in 1 the staining pattern was Grade A with HMFG2 only. Fifteen cases were Bloom's grade 3 and 66 were Bloom's grade 2 (Table III). No significant association was found between the staining grades A, B or 0 and Bloom's grade 2 or 3 (Chi-squared test). The lack of correlation was obtained in sections stained with either HMFG1 or HMFG2. ($P=7.23$; $P=6.77$).

**Figure 7** Relation of the staining grades to oestrogen receptor status. (□) = E.R.+; (■) = E.R.–.

**Table III** Relationship of the staining grades obtained with HMFG1 and HMFG2 to Bloom's Grade in 93 breast carcinomas

| | *Bloom's Grade* | | | | | |
|---|---|---|---|---|---|---|
| | *HMFG1* | | | *HMFG2* | | |
| *Staining grade* | *1* | *2* | *3* | *1* | *2* | *3* |
| A | 9 | 26 | 7 | 10 | 30 | 6 |
| B | 2 | 30 | 4 | 1 | 28 | 7 |
| O | 1 | 10 | 4 | 1 | 8 | 2 |

*Relation of the staining grades to oestrogen receptor status*

Twenty-four of the 56 cases from 1982–3 were oestrogen receptor positive and 32 were oestrogen receptor negative. There was no significant association between the staining grade A, B or 0 and the oestrogen receptor status of the tumours (Chi-squared test) (Figure 7). The lack of correlation was present in sections stained with either HMFG1 or HMFG2. ($P=1.93$, $P=2.04$).

*Relation of the staining grades to the presence of nodal metastases*

Thirty-five of the 89 cases were reported as having metastases in the axillary nodes and 55 had no nodal metastases. There is no significant association between the staining grade A, B or 0 and the presence or absence of nodal metastases (Chi-squared test) (Figure 8). The correlation was insignificant in sections stained with either HMFG1 or HMFG2 ($P=3.09$; $P=0.92$).

*Life table analysis comparing the staining grades*

Survival curves of patients from 1974–5 with staining grade A, B and 0 are illustrated in Figure 9. Although there appeared to be some differences between the curves, particularly with HMFG2, statistical analysis of the 3 curves showed that there was no significant difference in the survival of each

**Figure 8** Relation of staining grades to the presence of nodal metastases. (□)=Node +; (■)=Node−.

**Figure 9** Life table analysis comparing the staining grades. (□)=Grade O; (△)=Grade A; (▲)=Grade B.

staining group in sections stained with either HMFG1 or HMFG2 ($P$=0.79; $P$=0.18).

### Discussion

The immunohistochemical staining patterns of the monoclonal antibodies HMFG1 and HMFG2 in breast tissue have been described previously (Arklie *et al.*, 1981). The patterns observed in this study are similar, that is, extracellular in the ducts and tubules of normal and benign tissue and variable in malignant tissue.

The heterogeneity of staining in malignant breast tissue was apparent both in the extent of intracellular staining and in the relative intensity of staining. In some cases extracellular staining was observed both in tubules and between cells. Staining of the intracytoplasmic vacuoles was also considered extracellular, as electron microscopy has shown that the surface of the inner membrane of the vacuole has features typical of the exterior surface of the cell membranes (Battifora, 1975). In other cases staining was stronger intracellularly distributed either towards the periphery of the cell or diffusely throughout the cytoplasm. Nine percent of the tumours did not stain with either HMFG1 or HMFG2 and a further 2% did not stain with one of the antibodies. Wilkinson *et al.* (1984) have noted a similar percentage of negatively staining tumours.

Caution should be exerted when comparing immunohistochemical staining characteristics between studies, since differences in tissue preparation and staining technique may be of importance (Brandtzaeg & Rognum, 1982).

The change in the immunohistochemical staining pattern from extracellular in normal and benign breast tissue to mixed with varying amounts of intracellular positivity in malignant breast tissue has been observed using other antibodies (Sloane & Ormerod, 1981; Foster *et al.*, 1982) and lectins (Franklin, 1983).

The staining grades were designed to reflect the immunohistochemical staining patterns observed, whilst being in a form which could be related to indices of prognosis and to survival. Since the staining pattern in normal, benign and structurally differentiated tumours (those with extensive tubule formation) was extracellular, and became more intracellular as structural differentiation was lost, the grading system took into account the relative intensity of extracellular and intracellular staining and also the number of cells with intracellular staining. The 20 control sections where 2 consecutive sections were stained on separate occasions showed the same staining pattern on both sections but the overall intensity of stain throughout the section varied. Little emphasis, therefore, was placed on the overall intensity of the stain. Heterogeneity was observed between different areas of a tissue section and was a limitation of the grading system, for one area of tissue with a less predominant staining pattern, and therefore not graded, might influence the survival of the patient unnoticed. This heterogeneity also made grading difficult in many cases, but when the sections were graded on 2 separate occasions 84% reproducibility was obtained. This method of grading the stains contrasts with that used in another study relating the immunohistochemical staining of HMFG1 to survival and to indices of prognosis (Wilkinson *et al.*, 1984), where the grading was based on the uniformity, extent and the overall intensity of the stain.

There was no significant correlation when the staining grades were related to indices of prognosis and relapse-free survival. A significant correlation might have been expected when relating the staining grades to Bloom's grade since tumours with extensive tubule formation had an extracellular staining pattern. Three measures of differentiation contribute to Bloom's Grade of which tubule formation is one. The other 2 factors might combine to put a tumour with much tubule formation into Bloom's grade 2. Since extracellular staining was also recognised in intercellular spaces and intracytoplasmic vacuoles as well as in tubules, significant correlation with Bloom's grade is unlikely.

There was no association of the staining grades with oestrogen receptor status. In addition no correlation of the staining grades with involvement of axillary lymph nodes was observed. There was, however, a trend, in that a Grade A staining pattern with both HMFG1 and HMFG2 was more frequently seen in patients without lymph node involvement.

For HMFG1 and HMFG2 to be useful in determining the prognosis of breast cancer patients, the different staining patterns observed should be either closely correlated to existing prognostic indicators or be clearly related to survival, even in a relatively small series of cases. Relating the relapse-free survival of 34 patients to the staining grade, although there appeared to be some difference between the survival curves of the 3 staining grades, statistical comparison of the curves showed that they were not significantly different when either HMFG1 or HMFG2 was used. The antibodies are therefore of little use in routine diagnosis. Similar results were obtained by Wilkinson *et al.* (1984) where there was no significant association of the staining patterns observed to indices of prognosis such as Bloom's grade, the presence of metastases in the axillary nodes and the oestrogen receptor status. However, these authors identified a group of patients whose tumours did not stain with HMFG1 and who had a particularly poor prognosis. High levels of extracellular staining were considered indicators of a good prognosis. Although the immunoperoxidase staining technique was different in some respects to the one used in this study, a comparison of the staining patterns observed using the 2 techniques in 10 cases showed agreement. The different results must therefore be either due to the different method of grading used or, in the case of those patients with negatively stained tumours, the small number of cases.

The results of this study show that with present methods available for grading staining patterns the immunohistochemical staining patterns of HMFG1 and HMFG2 in malignant breast tissue do not help in determining the overall prognosis in an indivudual patient.

We acknowledge the work of the technical staff in the Pathology Dept., Faculty of Medicine, University of Southampton in preparing the illustrations.

**References**

ARKLIE, J., TAYLOR-PAPADIMITRIOU, J., BODMER, W., EGAN, M. & MILLIS, R. (1981). Differentiation antigens expressed by epithelial cells in the lactating breast are also detectable in breast cancers. *Int. J. Cancer*, **28**, 23.

BATTIFORA, H. (1975). Intracytoplasmic lumina in breast carcinoma. *Arch. Pathol.*, **99**, 614.

BLOOM, H.J.G. & RICHARDSON, W.W. (1957). Histological grading and prognosis in breast cancer. *Br. J. Cancer*, **11**, 359.

BRANDTZAEG, P. & ROGNUM, T.O. (1982). Evaluation of tissue preparation methods and paired immunofluorescence staining for immunocytochemistry of lymphomas. *Histochem. J.*, **15**, 655.

BURNS, J. (1978). Immunohistochemical methods and their application in the routine laboratory. In *Recent Advances In Histopathology*. p. 337. (Eds. Anthony & Woolf) Churchill & Livingstone.

CERIANI, R.L., THOMPSON, K.E., PETERSON, J.A. & ABRAHAM, S. (1977). Surface differentiation antigens on human mammary epithelial cells carried on the human milk fat globule. *Proc. Natl. Acad. Sci.*, **74**, 582.

CERIANI, R.L., SASAKI, M., SUSSMAN, H., WARA, W.M. & BLANK, E.W. (1982). Circulating human mammary epithelial antigens in breast cancer. *Proc. Natl Acad. Sci.*, **79**, 5420.

COOKE, T., GEORGE, D., SHIELDS, R., MAYNARD, P. & GRIFFITHS, K. (1979). Oestrogen receptors and prognosis in early breast cancer. *Lancet*, **i**, 995.

DEARNALEY, D.P., SLOANE, J.P., ORMEROD, M.G. & 6 others. (1981). Increased detection of mammary carcinoma cells in marrow smears using antisera to epithelial membrane antigen. *Br. J. Cancer*, **44**, 85.

FOSTER, C.S., DINSDALE, E.A., EDWARDS, P.A.W. & NEVILLE, A.M. (1982). Monoclonal antibodies to the human mammary gland II. *Virchows Arch.* (*Pathol. Anat.*), **394**, 295.

FRANKLIN, W.A. (1983). Tissue binding of lectins in disorders of the breast. *Cancer*, **51**, 295.

HEYDERMAN, E., STEELE, K. & ORMEROD, M.G. (1979). A new antigen on the epithelial membrane: its immunoperoxidase localisation in normal and neoplastic tissues. *J. Clin. Pathol.*, **32**, 35.

LEE, E., & DESU, M. (1972). A computer program for estimating survival functions for the life-table. *Comput Progr Biomed.*, **2**, 315.

MEPHAM, B.L., FRATER, W. & MITCHELL, B.S. (1979). The use of proteolytic enzymes to improve immunoglobulin staining by the PAP technique. *Histochem. J.*, **11**, 345.

NIE, N.H., HILL, C.H., JENKINS, J.C., STEINBRENNER, K. & BENT, D.H. (1981). *SPSS Updates 7–9*, McGraw Hill, New York.

ORMEROD, M.G., STEELE, K., EDWARDS, P.A.W. & TAYLOR-PAPADIMITRIOU, J. (1983). Monoclonal antibodies which react with epithelial membrane. (In press).

SLOANE, J.P., ORMEROD, M.G., IMRIE, S.F. & COOMBES, R.C. (1980). The use of antisera to epithelial membrane antigen in detecting micrometastases in histological sections. *Br. J. Cancer,* **42,** 392.

SLOANE, J.P. & ORMEROD, M.G. (1981). Distribution of epithelial membrane antigen in normal and neoplastic tissues and it's value in diagnostic tumour pathology. *Cancer,* **47,** 1786.

TAYLOR-PAPADIMITRIOU, J., PETERSON, J.A., ARKLIE, J., BURCHELL, J., CERIANI, R.L. & BODMER, W.F. (1981). Monoclonal antibodies to epithelium specific components of the milk fat globule membrane: production and reaction with cells in culture. *Int. J. Cancer,* **28,** 17.

TO, A., DEARNALEY, D.P., ORMEROD, M.G., CANTI, G. & COLEMAN, D.V. (1982). Epithelial membrane antigen. Its use in the cytodiagnosis of malignancy in serous effusions. *Am. J. Clin. Pathol.,* **78,** 214.

VALAGUSSA, P., BONADONNA, G. & VERONESI, U. (1978). Patterns of relapse and survival in operable breast cancer with positive and negative axillary nodes. *Tumori,* **64,** 241.

WILKINSON, M.J.S., HOWELL, A., HARRIS, M., TAYLOR-PAPADIMITRIOU, J., SWINDELL, R. & SELLWOOD, R.A. (1984). The prognostic significance of antigens expressed by human mammary tumour cells. *Int. J. Cancer,* **33,** 299.